# 超高效液相色谱-串联质谱法测定植物源性食品中环氧虫啶和哌虫啶残留

**DOI:** 10.3724/SP.J.1123.2024.04016

**Published:** 2025-03-08

**Authors:** Junjun LIU, Ju LI, Wanwan YU, Ying HAN, Xinxin MA, Chunrui ZHAN, Shixiang LI, Huawen WU, Kui HU, Jianchun WAN

**Affiliations:** 1.南昌海关技术中心, 江西 南昌 330038; 1. Technology Center of Nanchang Customs District, Nanchang 330038, China; 2.广州海关技术中心, 广东 广州 510623; 2. Technology Center of Guangzhou Customs District, Guangzhou 510623, China

**Keywords:** 超高效液相色谱-串联质谱法, 环氧虫啶, 哌虫啶, 植物源性食品, ultra performance liquid chromatography-tandem mass spectrometry, cycloxaprid, paichongding, foods of plant origin

## Abstract

本研究建立了超高效液相色谱-串联质谱法同时检测植物源性食品中环氧虫啶和哌虫啶2个非对映异构体残留量的分析方法,优化了干制水果和茶叶等基质的提取方式,确定了净化填料C_18_、PSA、GCB和无水硫酸镁的最佳用量。样品经乙腈提取,氯化钠盐析分层,采用基质分散固相萃取净化,经ACQUITY UPLC BEH C_18_色谱柱(100 mm×2.1 mm, 1.7 μm)分离,外标法定量。结果表明:环氧虫啶和哌虫啶在各自范围内呈良好的线性关系,相关系数均大于0.99,该方法的定量限为0.01 mg/kg,满足GB 2763-2021和GB 2763.1-2022规定的限量要求。用建立的方法对植物源性食品(稻谷、糙米、小麦、大米、花生、葡萄干、甘蓝、生菜、四季豆、西红柿、土豆、香菇、苹果、柑橘和茶叶等)进行加标回收验证,结果表明:15种基质在定量限1倍、2倍、10倍或GB 2763限量水平的加标平均回收率为78%~110%, RSD为0~12.8%。用本方法对100批果蔬样品进行分析检测,结果显示,环氧虫啶和哌虫啶均未检出。该方法前处理简单,普适性强,可作为植物源性食品中环氧虫啶和哌虫啶的确证定量检测方法。

## Abstract (en)

Neonicotinoid insecticides play an important role in the prevention and control of pests in crops, such as rice, wheat, corn, and vegetables, because of their broad spectrum, high efficiency, low toxicity, and low residue formation. However, their widespread use poses potential threats to the environment and human health. Cycloxaprid and paichongding are two new classes of neonicotinoid insecticides. The National Food Safety Standard Maximum Residue Limits for Pesticides in Foods (GB 2763) specifies the maximum residue limits for paichongding and cycloxaprid in rice, brown rice, wheat, and cabbage. However, the established limits are only temporary. In addition, no detection standards have yet been specified, and no relevant standards have been established in China. Therefore, establishing a method for analyzing cycloxaprid and paichongding residues in food is of particular importance. In this study, an optimized method was established to determine the residues of cycloxaprid and two noncorresponding isomers of paichongding in plant foods using ultra performance liquid chromatography-tandem mass spectrometry. The chromatographic conditions, matrix extraction methods for dried fruits and tea, and amounts of the purification materials C_18_, PSA, GCB, and anhydrous magnesium sulfate were optimized. The pesticides were extracted using an acetonitrile solution and separated from water with the addition of sodium chloride. The samples were centrifuged at 5000 r/min for 5 min, after which 1.00 mL of the supernatant was purified using 150 mg of anhydrous magnesium sulfate, 25 mg of C_18_, 25 mg of PSA, and 10 mg of GCB. Cycloxaprid and paichongding were separated on a ACQUITY UPLC BEH C_18_ chromatographic column (100 mm×2.1 mm, 1.7 μm) via gradient elution with 0.1% formic acid aqueous solution containing 5 mmol/L ammonium formate-acetonitrile as the mobile phase, and detected in electrospray positive ionization mode coupled with multiple-reaction monitoring (MRM) mode. Quantitative analysis was performed using an external standard method. Cycloxaprid and paichongding demonstrated good linear relationships within their respective concentration ranges, and the corresponding correlation coefficients were all above 0.99. The limits of detection (*S/N*=3) and quantification were 0.05 μg/kg and 0.01 mg/kg, respectively, which meet the requirements specified in GB 2763-2021 and GB 2763.1-2022. The established method was used to validate the recoveries of cycloxaprid and paichongding spiked in foods of plant origin, such as paddy, brown rice, wheat, rice, peanut, raisin, cabbage, lettuce, green bean, tomato, potato, shiitake mushroom, apple, citrus, and tea substrates, at three levels. The spiked levels covered the limit levels specified in GB 2763-2021 and GB 2763.1-2022. The mean recoveries of the target substances added to the 15 substrates at concentrations of 1, 2, and 10 times the LOQ or the limit values specified in GB 2763-2021 and GB 2763.1-2022 were distributed between 78% and 110%, and the RSDs were within the range of 0-12.8%. One hundred batches of fruit and vegetable samples were analyzed using the developed method, and the results showed that the selected samples were not contaminated with either cycloxaprid or paichongding. The proposed method is simple, sensitive, and universal, and has wide coverage. Thus, it can be used as a confirmatory and quantitative detection method for epoxidine and piperidine in foods of plant origin.

环氧虫啶(cycloxaprid)和哌虫啶(paichongding)是一类新烟碱杀虫剂^[1,2]^,具有广谱、高效、低毒、低残留等优点,对蚜虫、飞虱、粉虱、鞘翅目、双翅目等害虫有非常优异的杀虫活性,其杀虫效果显著优于吡虫啉、吡蚜酮和啶虫脒等农药,主要用于水稻、小麦、玉米、蔬菜等农作物中害虫的防治^[3]^。我国GB 2763-2021^[4]^和GB 2763.1-2022^[5]^《食品中农药最大残留限量标准》中规定了环氧虫啶和哌虫啶在稻谷、糙米、小麦以及甘蓝中的最大残留限量,均为临时限量,但并未指定检测标准,且国内外无相关检测标准。

哌虫啶含有2个手性中心,包括4个立体异构体:(5*R*,7*R*)-哌虫啶、(5*S*,7*S*)-哌虫啶、(5*S*,7*R*)-哌虫啶、(5*R*,7*S*)-哌虫啶,并以2对非对映异构体((5*R*,7*R*)-哌虫啶和(5*S*,7*S*)-哌虫啶、(5*S*,7*R*)-哌虫啶和(5*R*,7*S*)-哌虫啶互为对映异构体)形式存在^[6]^。目前国内外文献是主要针对环氧虫啶和哌虫啶在环境、土壤中降解以及防虫效果方面的研究^[7⇓-9]^,而针对食品中环氧虫啶和哌虫啶残留量检测方法研究较少。张艳等^[10]^利用超高效液相色谱-串联质谱法,建立了蔬菜中环氧虫啶等残留物质的检测方法。王霞等^[11]^采用分散固相萃取结合超高效液相色谱-串联质谱法建立了蔬菜中环氧虫啶等10种新烟碱类农药残留的检测方法。丛路静等^[12]^分别利用二氯甲烷和乙腈提取,C_18_固相萃取小柱净化,建立了高效液相色谱分析稻田水、糙米和稻壳中哌虫啶残留量的方法。文献调研结果显示,环氧虫啶和哌虫啶同时分析检测的报道较少,庄鹏等^[13]^结合QuEChERS,建立了高效液相色谱-串联质谱法同时检测环氧虫啶和哌虫啶的方法,所测基质为绿橙、荔枝、燕窝果、红毛丹等热带水果,无法覆盖国内流通量大的茶叶、稻谷等基质。本研究采用超高效液相色谱-串联质谱检测技术,结合QuEChERS,建立了谷物、坚果、干制水果、果蔬、茶叶等基质中环氧虫啶和哌虫啶同时分析检测的方法,15种基质中方法最高检出限为0.05 μg/kg,其中苹果中哌虫啶检出限低至0.007 μg/kg。该方法前处理简单,普适性强,可为食品安全风险监测提供检测依据。

## 1 实验部分

### 1.1 仪器与试剂

U3000-TSQ Quantiva型超高效液相色谱-串联质谱仪(美国Thermo Fisher公司); IKA KS 4000ic控温摇床(德国IKA公司); BSA2202S型电子天平(德国Sartorius公司); ME155DU型电子天平(瑞士Mettler Toledo公司); TDL-5-A型离心机(上海安亭科学仪器厂); ELGA Q15型超纯水装置(英国ELGA公司)。

环氧虫啶标准品(CAS号:1203791-41-6)、(5*R*,7*S*)-哌虫啶标准品(CAS号:1214742-91-2)、(5*R*,7*R*)-哌虫啶标准品(CAS号:1213785-98-8),纯度均>99.0%,天津阿尔塔科技有限公司。

乙腈(色谱纯,德国Merck公司);氯化钠(分析纯,西陇科学股份有限公司);无水硫酸镁(分析纯,天津市大茂化学试剂厂,500 ℃灼烧4 h,置于干燥器中保存); *N*-丙基乙二胺吸附剂(PSA,粒径40~63 μm,上海安谱实验科技股份有限公司);十八烷基键合硅胶吸附剂(C_18_,粒径40~60 μm)、石墨化炭黑(GCB,粒径40~120 μm)(天津博纳艾杰尔科技有限公司)。

### 1.2 标准溶液的配制

分别精确称取环氧虫啶和哌虫啶标准品10 mg,用乙腈溶解定容至10 mL,配制成质量浓度为1.0 mg/mL的标准储备溶液,使用前用乙腈稀释成适当质量浓度的标准中间液。

### 1.3 样品前处理

谷物油料基质:称取粉碎后的稻谷、糙米、小麦、大米、花生试样各2.5 g,分别加入7.5 mL水,涡旋分散,静置30 min;干制水果基质:称取粉碎后的葡萄干试样2.5 g,加入7.5 mL水,均质分散;果蔬基质:称取匀浆后的甘蓝、生菜、四季豆、西红柿、土豆、香菇、苹果、柑橘试样各10 g;特殊样品基质:称取粉碎后的茶叶试样2.0 g。向上述样品中加入10 mL乙腈,涡旋分散,振荡提取5 min,加入5 g氯化钠快速混匀(茶叶基质无需加氯化钠),于5000 r/min离心5 min,上清液待净化。

吸取1.00 mL上清液至聚丙烯离心管中(内含150 mg无水硫酸镁、25 mg C_18_、25 mg PSA、10 mg GCB),涡旋混合30 s,于5000 r/min离心5 min,准确移取上清液0.50 mL,加入0.50 mL水,混匀后过0.22 μm微孔滤膜,上机测定。

### 1.4 分析条件

#### 1.4.1 色谱条件

色谱柱:ACQUITY UPLC BEH C_18_色谱柱(100 mm×2.1 mm, 1.7 μm,美国Waters公司);流动相A:乙腈,流动相B: 0.1%甲酸水溶液(含5 mmol/L甲酸铵)。梯度洗脱:0~1.0 min, 10%A; 1.0~4.0 min, 10%A~50%A; 4.0~5.5 min, 50%A~95%A; 5.5~6.5 min, 95%A; 6.5~6.51 min, 95%A~10%A; 6.51~9.0 min, 10%A。柱温:35 ℃;流速:0.3 mL/min;进样量:5 μL。

#### 1.4.2 质谱条件

离子源:ESI源,正离子模式;电喷雾电压:3500 V;离子源温度:400 ℃;离子传输管温度:350 ℃;鞘气流量: 40 Arb;雾化气流量: 10 Arb;反吹气流量: 1 Arb;碰撞气:高纯氩气;碰撞气压力:0.2 Pa。多反应监测模式(MRM)扫描,其他质谱参数见表1。

**表1:** 环氧虫啶、(5*R*,7*S*)-哌虫啶、(5*R*,7*R*)-哌虫啶质谱参数

Compound	Precursor ion (m/z)	Product ion (m/z)	RF lens/V	Collision energy/eV
Cycloxaprid	323.1	126.0^*^	80	46
		277.1		15
(5R,7S)-	367.2	263.1^*^	79	17
Paichongding		321.2		12
(5R,7R)-	367.2	263.1^*^	79	17
Paichongding		321.2		12

RF lens: radio frequency lens; * quantitative ion.

## 2 结果与讨论

### 2.1 色谱条件的优化

流动相采用0.1%甲酸水溶液和乙腈,调节不同梯度洗脱条件,对比了Waters ACQUITY UPLCBEH C_18_(100 mm×2.1 mm, 1.7 μm)和Thermo Hypersil GOLD C_18_(100 mm×2.1 mm, 1.9 μm)两款色谱柱对环氧虫啶和哌虫啶的分离效果。结果表明:环氧虫啶在两款色谱柱上分离效果较为接近,哌虫啶均未达到基线分离,但经Waters ACQUITY UPLC BEH C_18_色谱柱分离的峰形更尖锐,因此方法确定采用Waters ACQUITY UPLC BEH C_18_色谱柱继续进行流动相的优化。

实验尝试调整流动相组成,具体如下:配制混合标准溶液0.5 mg/L(定容液为50%乙腈水溶液),分别用0.1%甲酸水(含2 mmol/L甲酸铵)-乙腈、0.1%甲酸水(含5 mmol/L甲酸铵)-乙腈、0.1%甲酸水(含10 mmol/L甲酸铵)-乙腈作为流动相进行分离。实验结果表明:流动相中加入甲酸铵后,哌虫啶两个异构体达到基线分离,甲酸铵浓度越高,分离度越好;环氧虫啶随着甲酸铵浓度的提高,峰形前沿现象改善明显,50%乙腈水溶液定容,流动相中含2 mmol/L的甲酸铵时,环氧虫啶双肩峰明显,流动相中甲酸铵浓度增至5 mmol/L时,环氧虫啶双肩峰消失,考虑盐浓度对方法灵敏度和仪器的影响,本方法确定流动相为0.1%甲酸水(含5 mmol/L甲酸铵)-乙腈。优化色谱条件下环氧虫啶和哌虫啶的提取离子色谱图如图1所示。

**图1:**
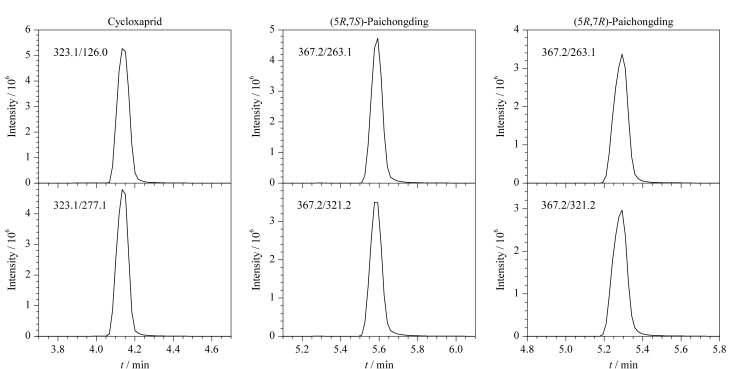
环氧虫啶、(5*R*,7*S*)-哌虫啶、(5*R*,7*R*)-哌虫啶的提取离子流色谱图

环氧虫啶极性稍强,受定容液中有机相比例影响较明显,实验取标准溶液(1 mg/L,乙腈作为定容液)适量,加水稀释,使得标准溶液和水的体积比分别为10∶0、9∶1、8∶2、7∶3、6∶4、5∶5、4∶6、3∶7、2∶8、1∶9,采用1.4节的分析条件对环氧虫啶进行分析。结果表明:当标准溶液体积小于水的体积时,环氧虫啶双肩峰和峰前沿情况消失,综合考虑色谱峰形和方法灵敏度,选择50%乙腈水溶液作为定容液。

### 2.2 前处理方法的优化

#### 2.2.1 提取条件的优化

本实验涉及的15种基质可分为含水量高的果蔬、含水量低的谷物、干制水果和茶叶基质。含水量高的果蔬基质,因水与乙腈互溶,此类基质的提取效果较好;含水量低的基质(如稻谷),加入乙腈后因吸湿性导致提取溶剂减少,高脂肪、高糖分样品黏性大,易结团,直接提取效果不理想,SANTE 11312/2021^[14]^中规定,为提高低水分样品基质(如谷物、干果等)的提取效率,需提取前在样品中加入水,因此本实验针对高脂肪基质花生、高糖分基质干果样品进行加水分散提取验证,采用静置振荡、均质方式进行比对,保证样品的分散性。实验方法如下:分别选择粉碎后的花生、葡萄干样品基质,按样品和水质量比为1∶3的比例加水,浸泡0、10、20、30 min,观察样品分散程度,再加入乙腈振荡提取5 min,观察试样提取后的分散程度。实验结果表明,花生基质随着浸泡时间延长,样品从结块、逐渐浸润分散,到30 min后涡旋呈完全分散状态,加入乙腈振荡提取5 min后,样品呈均一状态;葡萄干样品随着浸泡时间的延长,样品逐渐扩散至水中,30 min后仍有部分结块样品,加入乙腈振荡提取5 min后,结块样品变少,但仍未完全分散;粉碎后的葡萄干样品按上述比例加水直接均质处理后,样品分散成均一状态。综上所述,花生和葡萄干基质分别采用加水静置和加水均质方式分散,再加入乙腈进行振荡提取,含水量少的谷物基质参照花生处理方式提取。

#### 2.2.2 净化条件的优化

log *P*值是指某物质在正辛醇和水体系分配系数的对数,禤学怡^[15]^和Toshio等^[16]^建议采用log *P*值作为药物的亲脂性参数,即log *P*值越大,该物质越亲脂。检索ChemSpider数据库可知环氧虫啶、哌虫啶的log *P*值分别为2.44和3.72,属于亲脂性化合物,参考现行相关标准及文献^[17⇓⇓⇓⇓⇓⇓⇓⇓⇓⇓⇓⇓⇓-31]^,本实验采用乙腈提取、基质分散固相萃取方式净化,净化填料选择范围包括C_18_、PSA、GCB、无水MgSO_4_,其组合方式及比例采用正交试验设计进行优化。选择典型基质西红柿(含水量大)、花生(含水量少,含油脂和淀粉)、茶叶(特殊样品)进行试验。前处理过程如下:称取茶叶粉末2.0 g,加入水8 mL,静置30 min,提取、净化操作同1.3节,西红柿和花生样品称量、提取、净化操作同1.3节,净化填料组成见表2,此时用溶剂标准溶液曲线进行定量。

**表2:** 净化填料组合的正交试验设计和极差分析结果

No.	Influence factors					Recoveries/%			
	C_18_/mg	PSA/mg	GCB/mg	MgSO_4_/mg		Tomatoes	Peanuts		Tea
Test 1 (cycloxaprid)	0	0	0	150		128	121		61.4
Test 1 (paichongding)						110	147		90.6
Test 2 (cycloxaprid)	0	25	10	300		128	167		60.3
Test 2 (paichongding)						115	149		82.2
Test 3 (cycloxaprid)	0	50	25	600		124	163		57.6
Test 3 (paichongding)						111	144		71.4
Test 4 (cycloxaprid)	25	0	10	600		117	111		65.1
Test 4 (paichongding)						107	136		88.7
Test 5 (cycloxaprid)	25	25	25	150		137	161		56.3
Test 5 (paichongding)						109	146		79.7
Test 6 (cycloxaprid)	25	50	0	300		145	155		56.4
Test 6 (paichongding)						111	143		76.2
Test 7 (cycloxaprid)	50	0	25	300		133	109		58.8
Test 7 (paichongding)						104	137		82.2
Test 8 (cycloxaprid)	50	25	0	600		141	154		56.9
Test 8 (paichongding)						107	139		78.6
Test 9 (cycloxaprid)	50	50	10	150		143	143		56.2
Test 9 (paichongding)						110	142		74.6
(cycloxaprid)/%	127^a^, 151^b^, 59.8^c^	126^a^, 114^b^, 61.8^c^	138^a^, 143^b^, 58.2^c^	136^a^, 142^b^, 58.0^c^					
(paichongding)/%	112^a^, 146^b^, 81.4^c^	107^a^, 140^b^, 87.2^c^	109^a^, 143^b^, 81.8^c^	110^a^, 145^b^, 81.6^c^					
(cycloxaprid)/%	133^a^, 142^b^, 59.3^c^	136^a^, 161^b^, 57.8^c^	130^a^, 141^b^, 60.5^c^	135^a^, 144^b^, 58.5^c^					
(paichongding)/%	109^a^, 142^b^, 81.5^c^	111^a^, 144^b^, 80.2^c^	111^a^, 142^b^, 81.8^c^	110^a^, 143^b^, 80.2^c^					
(cycloxaprid)/%	139^a^, 135^b^, 57.3^c^	138^a^, 154^b^, 56.7^c^	132^a^, 144^b^, 57.6^c^	128^a^, 143^b^, 59.9^c^					
(paichongding)/%	107^a^, 139^b^, 78.5^c^	111^a^, 143^b^, 74.1^c^	108^a^, 142^b^, 77.8^c^	109^a^, 140^b^, 79.6^c^					
R (cycloxaprid)/%	12.4^a^, 15.2^b^, 2.47^c^	11.3^a^, 47.0^b^, 5.03^c^	8.50^a^, 3.80^b^, 3.00^c^	8.60^a^, 2.00^b^, 1.90^c^					
R (paichongding)/%	4.63^a^, 7.40^b^, 3.07^c^	3.83^a^, 4.33^b^, 13.1^c^	2.77^a^, 0.633^b^, 4.07^c^	1.70^a^, 4.83^b^, 2.07^c^					

a: tomatoes; b: peanuts; c: tea.

正交试验极差分析结果(表2)表明,环氧虫啶在西红柿、花生中回收率普遍偏高,C_18_和PSA对回收率影响稍明显,尤其是花生基质中PSA对回收率有显著影响,C_18_和PSA用量较低时回收率更好,茶叶基质中回收率则偏低,且定性定量离子受基质干扰明显,影响结果的准确性;哌虫啶在西红柿中回收率正常,在花生中回收率较高,C_18_用量对其影响较显著,茶叶的回收率则略低,PSA用量对其回收率影响较明显。

表2中,GCB、无水MgSO_4_在西红柿基质中对环氧虫啶虽不是最显著的影响因子(*R*),但值均在8以上,说明用量对回收率也有影响。

综合考虑,确定用25 mg C_18_、25 mg PSA、10 mg GCB、150 mg无水MgSO_4_对1 mL提取样液进行净化。

茶叶中环氧虫啶和哌虫啶受基质干扰严重,其干扰物可能来自水浸泡后的萃取物。实验设计对比茶叶称量后加水静置再乙腈提取和称量后直接乙腈提取,净化等步骤见1.3节。实验结果表明,乙腈直接提取,消除了环氧虫啶和哌虫啶定性定量离子受干扰的情况,说明茶叶加入水后,乙腈萃取的极性干扰物更多,对极性稍强的环氧虫啶形成了干扰。最终确定茶叶不加水直接用乙腈提取。

### 2.3 基质效应

质谱离子化效率受基体成分的影响,导致的基质效应(ME)可能对定量结果产生影响,本实验选择稻谷、茶叶、甘蓝、柑橘等典型样品基质进行了基质效应评价,实验方法如下:按本实验确定的样品前处理方法,制备稻谷、茶叶、甘蓝、柑橘空白基质溶液,配制系列基质标准溶液,同时用50%乙腈水溶液配制系列溶剂标准溶液,上机测定,分别拟合基质标准工作曲线和溶剂标准工作曲线,计算ME。ME=(基质匹配校准曲线斜率/溶剂标准曲线斜率)×100%。当ME为80%~120%为弱基质效应,ME为50%~80%或者120%~150%时为中等基质效应,ME≤50%或≥150%时为强基质效应^[32,33]^。

实验结果表明:环氧虫啶、(5*R*,7*S*)-哌虫啶和(5*R*,7*R*)-哌虫啶在稻谷、茶叶、甘蓝、柑橘样品中呈现基质效应不同,强度有差异,部分项目和基质效应远超80%~120%的要求(见表3),因此本实验确定采用基质标准曲线定量。

**表3:** 环氧虫啶、(5*R*,7*S*)-哌虫啶和(5*R*,7*R*)-哌虫啶在稻谷、茶叶、甘蓝、柑橘样品中的基质效应

Compound	MEs/%			
	Paddy	Tea	Cabbage	Citrus
Cycloxaprid	758	90	86	65
(5R,7S)-Paichongding	819	89	92	81
(5R,7R)-Paichongding	893	90	113	92

### 2.4 方法学评价

#### 2.4.1 线性范围与检出限

取1.2节标准工作液适量,用空白基质提取溶液配制成不同质量浓度的基质混合标准工作溶液,在1.4节的条件下,对基质混合标准工作溶液进样测定,以峰面积为纵坐标,目标物质量浓度为横坐标绘制标准工作曲线,得到线性方程和相关系数。结果表明:15种基质样品中环氧虫啶和哌虫啶在各自的线性范围内线性相关性良好,线性相关系数>0.99。在空白样品中添加标准溶液,制得加标样品,以加标样品中测得目标物信噪比(*S/N*)等于3时的含量确定方法的检出限(LOD),为0.05 μg/kg(以茶叶基质代表);在满足GB 2763-2021和GB 2763.1-2022的限量检测要求下,方法的定量限为0.01 mg/kg,能充分适合各型号液相色谱-串联质谱仪的检测。15种基质中目标物的线性范围、线性方程等见表4。

**表4:** 15种基质中环氧虫啶和哌虫啶的线性范围、线性方程、加标回收率和相对标准偏差(*n*=6)

Matrix	Compound	Linear range/ (μg/L)	Linear equation	Spiked/(mg/kg)	Recoveries/%	RSDs/%
Paddy	cycloxaprid	0.5-20.0	Y=27650X-772.2	0.01, 0.02, 0.1	88.2, 90.8, 99.0	4.73, 4.14, 6.76
	(5R,7S)-paichongding	2.0-100.0	Y=39400X+14720	0.01, 0.02, 0.5	94.8, 95.8, 100	3.91, 2.13, 2.65
	(5R,7R)-paichongding	2.0-100.0	Y=27660X+9396	0.01, 0.02, 0.5	94.7, 95.0, 101	3.18, 0, 1.08
Brown rice	cycloxaprid	0.5-20.0	Y=28000X+347.2	0.01, 0.02, 0.1	80.2, 85.8, 97.3	2.67, 4.39, 4.29
	(5R,7S)-paichongding	2.0-100.0	Y=40090X-296.3	0.01, 0.02, 0.5	101, 96.7, 100	3.76, 4.22, 2.33
	(5R,7R)-paichongding	2.0-100.0	Y=28120X-1978	0.01, 0.02, 0.5	97.0, 96.7, 102	3.45, 2.67, 2.48
Wheat	cycloxaprid	0.5-20.0	Y=40550X-1487	0.01, 0.02, 0.1	93.0, 93.3, 100	4.08, 5.53, 5.48
	(5R,7S)-paichongding	0.5-20.0	Y=49730X-255.8	0.01, 0.02, 0.1	103, 103, 103	5.00, 2.50, 7.74
	(5R,7R)-paichongding	0.5-20.0	Y=37120X-2044	0.01, 0.02, 0.1	103, 102, 105	5.00, 2.67, 5.22
Rice	cycloxaprid	0.5-20.0	Y=42850X-2430	0.01, 0.02, 0.1	96.0, 96.7, 97.8	5.47, 7.07, 3.79
	(5R,7S)-paichongding	0.5-20.0	Y=51990X-4239	0.01, 0.02, 0.1	104, 102, 104	6.11, 4.08, 6.66
	(5R,7R)-paichongding	0.5-20.0	Y=38650X-3893	0.01, 0.02, 0.1	105, 102, 108	5.72, 2.54, 3.77
Cabbage	cycloxaprid	20.0-1000.0	Y=12870X+179800	0.01, 0.02, 1.0	85.0, 85.0, 88.5	6.47, 4.80, 4.21
	(5R,7S)-paichongding	2.0-100.0	Y=41000X+3059	0.01, 0.02, 0.1	100, 100, 110	6.01, 2.58, 3.71
	(5R,7R)-paichongding	2.0-100.0	Y=31190X+1515	0.01, 0.02, 0.1	107, 105, 110	4.83, 1.94, 4.69
Lettuce	cycloxaprid	2.0-100.0	Y=33740X+11180	0.01, 0.02, 0.1	78.0, 80.0, 84.0	11.3, 9.41, 5.15
	(5R,7S)-paichongding	2.0-100.0	Y=46650X+16280	0.01, 0.02, 0.1	89.0, 90.0, 90.0	10.3, 6.09, 6.15
	(5R,7R)-paichongding	2.0-100.0	Y=35340X+6440	0.01, 0.02, 0.1	98.0, 105, 100	10.0, 3.98, 0.410
Green beans	cycloxaprid	2.0-100.0	Y=27030X+28060	0.01, 0.02, 0.1	88.0, 85.0, 85.0	12.8, 11.8, 11.2
	(5R,7S)-paichongding	2.0-100.0	Y=39070X+8445	0.01, 0.02, 0.1	103, 100, 90.0	7.93, 10.4, 7.73
	(5R,7R)-paichongding	2.0-100.0	Y=47190X+45800	0.01, 0.02, 0.1	102, 100, 100	4.00, 2.58, 1.10
Tomatoes	cycloxaprid	2.0-100.0	Y=31030X+18740	0.01, 0.02, 0.1	86.0, 90.0, 91.0	3.87, 4.18, 3.92
	(5R,7S)-paichongding	2.0-100.0	Y=38840X+16750	0.01, 0.02, 0.1	103, 100, 100	5.95, 6.65, 5.04
	(5R,7R)-paichongding	2.0-100.0	Y=29760X+7596	0.01, 0.02, 0.1	107, 105, 110	4.83, 4.26, 5.75
Potatoes	cycloxaprid	2.0-100.0	Y=32970X+13220	0.01, 0.02, 0.1	97.0, 85.0, 91.0	7.57, 4.80, 7.67
	(5R,7S)-paichongding	2.0-100.0	Y=45520X+1512	0.01, 0.02, 0.1	100, 95.0, 100	0, 4.40, 8.96
	(5R,7R)-paichongding	2.0-100.0	Y=35240X-1882	0.01, 0.02, 0.1	103, 95.0, 100	5.01, 8.81, 3.35
Shiitake mushrooms	cycloxaprid	2.0-100.0	Y=28550X+10500	0.01, 0.02, 0.1	82.0, 85.0, 86.0	4.61, 3.04, 2.37
	(5R,7S)-paichongding	2.0-100.0	Y=39680X+6517	0.01, 0.02, 0.1	101, 110, 100	5.38, 2.49, 6.65
	(5R,7R)-paichongding	2.0-100.0	Y=30800X+1600	0.01, 0.02, 0.1	102, 100, 100	4.00, 4.08, 5.89
Apples	cycloxaprid	2.0-100.0	Y=37120X+10770	0.01, 0.02, 0.1	91.0, 90.0, 89.0	4.02, 3.51, 2.40
	(5R,7S)-paichongding	2.0-100.0	Y=48190X+4340	0.01, 0.02, 0.1	101, 95.0, 100	7.99, 6.15, 1.63
	(5R,7R)-paichongding	2.0-100.0	Y=37280X-4100	0.01, 0.02, 0.1	103, 105, 110	11.8, 3.58, 4.98
Citrus	cycloxaprid	2.0-100.0	Y=34250X+5456	0.01, 0.02, 0.1	88.0, 90.0, 88.0	4.64, 4.18, 4.03
	(5R,7S)-paichongding	2.0-100.0	Y=44810X+13580	0.01, 0.02, 0.1	103, 100, 100	5.01, 5.16, 4.75
	(5R,7R)-paichongding	2.0-100.0	Y=35520X+10500	0.01, 0.02, 0.1	105, 105, 100	5.22, 3.58, 5.31
Peanuts	cycloxaprid	0.5-20.0	Y=41570X-3084	0.01, 0.02, 0.1	96.0, 100, 98.0	4.10, 3.76, 2.08
	(5R,7S)-paichongding	0.5-20.0	Y=47450X-8206	0.01, 0.02, 0.1	105, 105, 100	5.22, 1.94, 4.08
	(5R,7R)-paichongding	0.5-20.0	Y=35400X-5972	0.01, 0.02, 0.1	103, 100, 100	5.01, 3.76, 6.12
Raisins	cycloxaprid	0.5-20.0	Y=43670X- 7243	0.01, 0.02, 0.1	93.0, 95.0, 95.0	4.70, 4.40, 9.38
	(5R,7S)-paichongding	0.5-20.0	Y=54870X-7728	0.01, 0.02, 0.1	98.0, 100, 100	8.24, 3.76, 4.67
	(5R,7R)-paichongding	0.5-20.0	Y=40680X-7354	0.01, 0.02, 0.1	104, 100, 100	6.82, 6.06, 4.52
Tea	cycloxaprid	0.5-20.0	Y=26040X+1008	0.01, 0.02, 0.1	89.0, 90.0, 83.0	5.98, 16.0, 11.7
	(5R,7S)-paichongding	0.5-20.0	Y=39000X-2424	0.01, 0.02, 0.1	91.0, 85.0, 80.0	9.32, 8.92, 4.84
	(5R,7R)-paichongding	0.5-20.0	Y=28160X-410	0.01, 0.02, 0.1	86.0, 85.0, 80.0	12.3, 9.67, 5.90

*Y*: peak area; *X*: mass concentration, μg/L.

#### 2.4.2 回收率与精密度

按本实验确定的样品前处理方法和分析条件,对环氧虫啶和哌虫啶在15种基质样品中进行三水平加标回收验证(*n*=6),加标水平涵盖GB 2763-2021和GB 2763.1-2022的限量水平,具体加标水平及结果见表4。实验结果表明:在1倍、2倍、10倍定量限添加水平下的平均回收率在78%~110%范围内,符合GB/T 27404-2008^[34]^中规定的要求(在0.01 mg/kg和0.02 mg/kg添加水平的加标样品平均回收率在60~120%范围内,在0.5 mg/kg和1.0 mg/kg添加水平的加标样品平均回收率在80%~110%范围内)。

### 2.5 实际样品的检测

用本实验建立的方法对来自江西省内不同区域的100批果蔬样品进行处理和分析,结果表明:实际样品中均未检出环氧虫啶和哌虫啶残留,可能和这两种农药的登记使用时间不长、应用不广泛有关。

## 3 结论

本研究建立了超高效液相色谱-串联质谱法定量分析植物源性食品中环氧虫啶和哌虫啶残留量的方法,探讨了植物源性食品中环氧虫啶、(5*R*,7*S*)-哌虫啶、(5*R*,7*R*)-哌虫啶残留量同时测定的色谱分离技术和各类植物源性食品前处理技术,所建立的方法选择性强,灵敏度高,能够满足GB 2763-2021和GB 2763.1-2022中规定的环氧虫啶和哌虫啶限量的检测要求,可为我国食品安全监管提供技术支持。

## References

[1] Nie H Y, Yang R B, Zhao Y L, et al. Chinese Journal of Pesticide Science, 2016, 18(3): 352

[2] Xu X Y, Shao X S, Wu C Y, et al. World Pestcides, 2009, 31(4): 52

[3] Zhang J B. [MS Dissertation]. Hangzhou: Zhejiang University, 2014

[4] GB 2763-2021

[5] GB 2763.1-2022

[6] Li J Y. [MS Dissertation]. Hangzhou: Zhejiang University, 2014

[7] Xie H, Zhu L S, Tan M Y. Acta Pedologica Sinica, 2016, 53(1): 232

[8] Zhang Y L, An G S, Liu B S, et al. Jiangsu Agricultural Sciences, 2021, 49(4): 66

[9] Huang Q C, Dai C C, Zhang J P, et al. Xinjiang Agricultural Sciences, 2020, 57(11): 2050

[10] Zhang Y, Qu L J, Ling L, et al. Modern Preventive Medicine, 2024, 51(4): 722

[11] Wang X, Zhang W Y, Wang M, et al. Chinese Journal of Analysis Laboratory, 2023, 42(7): 897

[12] Cong L J, Liu J S, Wang M Y, et al. Journal of Food Safety and Quality, 2014, 5(3): 912

[13] Zhuang P, Ji S F, Xie H Y, et al. China Port Science and Technology, 2023, 5(3): 66

[14] SANTE 11312/2021

[15] Xuan X Y. [MS Dissertation]. Guangzhou: Guangdong Pharmaceutical University, 2015

[16] Toshio F, Junkichi I, Corwin H. J Am Chem Soc, 1964, 86(23): 5175

[17] GB 23200.121-2021

[18] GB 23200.113-2018

[19] EN 15662: 2018

[20] Kardani F, Jelyani A Z, Hashemi M, et al. J Food Compos Anal, 2023: 124

[21] Song W, Liu K Y, Chen L J, et al. Chinese Journal of Chromatography, 2024, 42(1): 52 38197206 10.3724/SP.J.1123.2023.04001 PMC10782281

[22] Li J, Ju X, Wang Y L, et al. Chinese Journal of Chromatography, 2023, 41(7): 610 37387282 10.3724/SP.J.1123.2022.10010 PMC10311622

[23] Xu R H, Xie Q W, Li X J, et al. Chinese Journal of Chromatography, 2022, 40(5): 469 35478006 10.3724/SP.J.1123.2021.11015 PMC9404200

[24] Yan X X, Tong K X, Zhu Z H, et al. Journal of Instrumental Analysis, 2024, 43(4): 590

[25] Peng J, Mu Y C, Yu Y L, et al. Food Science, 2024, 45(3): 185

[26] Zhang J W, Wang Y H, Liu J N, et al. Chinese Journal of Analytical Chemistry, 2023, 51(11): 1802

[27] Zhang S P, Zhou J. Journal of Instrumental Analysis, 2023, 42(10): 1301

[28] Yuan H F, Song S W, Gao H W, et al. China Tea, 2023, 45(8): 39

[29] Li J, Wang Y L, Li F F, et al. Chinese Journal of Analytical Chemistry, 2023, 51(10): 1693

[30] Xue X H, Li T T, Liu P, et al. Science and Technology of Food Industry, 2023, 44(4): 337

[31] Liu J J, Wang L M. China Food Additives, 2023, 34(5): 274

[32] Tong L Y, Xu B Z, Nie X M, et al. Chinese Journal of Chromatography, 2023, 41(11): 986 37968817 10.3724/SP.J.1123.2023.07010 PMC10654881

[33] Sapozhnikova Y, Lehotay S J. Anal Chim Acta, 2013, 758: 80 23245899 10.1016/j.aca.2012.10.034

[34] GB/T 27404-2008

